# Pikachurin Protein Required for Increase of Cone Electroretinogram B-Wave during Light Adaptation

**DOI:** 10.1371/journal.pone.0128921

**Published:** 2015-06-19

**Authors:** Masatoshi Nagaya, Shinji Ueno, Taro Kominami, Ayami Nakanishi, Toshiyuki Koyasu, Mineo Kondo, Takahisa Furukawa, Hiroko Terasaki

**Affiliations:** 1 Department of Ophthalmology, Nagoya University Graduate School of Medicine, Nagoya, Aichi, Japan; 2 Department of Ophthalmology, Mie University Graduate School of Medicine, Tsu, Mie, Japan; 3 Laboratory for Molecular and Developmental Biology, Institute for Protein Research, Osaka University, Suita, Osaka, Japan; 4 JST, CREST, Suita, Osaka, Japan; Dalhousie University, CANADA

## Abstract

In normal eyes, the amplitude of the b-wave of the photopic ERGs increases during light adaptation, but the mechanism causing this increase has not been fully determined. The purpose of this study was to evaluate the contribution of receptoral and post-receptoral components of the retina to this phenomenon. To accomplish this, we examined the ERGs during light adaptation in Pikachurin null-mutant (Pika -/-) mice, which have a misalignment of the bipolar cell dendritic tips to the photoreceptor ribbon synapses. After dark-adaptation, photopic ERGs were recorded from Pika -/- and wild type (WT) mice during the first 9 minutes of light adaptation. In some of the mice, post-receptoral components were blocked pharmacologically. The photopic b-waves of WT mice increased by 50% during the 9 min of light adaptation as previously reported. On the other hand, the b-waves of the Pika -/- mice decreased by 20% during the same time period. After blocking post-receptoral components, the b-waves were abolished from the WT mice, and the ERGs resembled those of the Pika -/- mice. The extracted post-receptoral component increased during light adaptation in the WT mice, but decreased for the first 3 minutes to a plateau in Pika -/- mice. We conclude that the normal synaptic connection between photoreceptor and retinal ON bipolar cells, which is controlled by pikachurin, is required for the ERGs to increase during light-adaptation. The contributions of post-receptoral components are essential for the photopic b-wave increase during the light adaptation.

## Introduction

Photopic electroretinograms (ERGs) are used to assess the functional properties of the cone system of patients with various types of retinal diseases. After dark-adaptation, the amplitudes of photopic ERG b-wave increase gradually during the early phase of light adaptation. Because of this, the International Society for Clinical Electrophysiology of Vision (ISCEV) recommends a minimum of 10 min of light adaptation before the recording of the photopic ERGs [1]. This increase has been documented in humans for more than 60 years [2–7], and it is also observed in fish [8] and rodents [9,10].

Several explanations have been suggested for this phenomenon [3–5,8,10–12]. One of the plausible mechanisms is that under dark-adapted conditions, the rods inhibit the cones, and under light-adapted conditions, this inhibition is released resulting in an increase in the cone ERGs [4,8,11]. For this rod inhibition, interactions of the post-receptoral neural elements including bipolar and horizontal cells [4,8,13] and direct interaction of cone and rod [11] were proposed. Another mechanism suggested was that the cone photoreceptor responses increase during light adaptation because the amplitudes of the photopic ERG a-waves also increase during the course of light adaptation [5,10]. In addition to these possibilities, several other mechanisms have been proposed [3,14], but some are inconsistent with others, and the mechanism of the increase of the amptitude of the photopic ERG b-wave remains undetermined.

Pikachurin null-mutant (Pika-/-) mice have been recently created. Pikachurin is an extracellular matrix-like protein located mainly in the synaptic cleft of the photoreceptors adjacent to the ribbon synapses. Pika-/- mice have a misaligned apposition of the bipolar cell dendritic tips to the photoreceptor ribbon synapses resulting in alterations of the synaptic signal transmission between the photoreceptors and bipolar cells [15,16]. The amplitudes of the b-waves of the photopic ERGs are reduced in Pika-/- mice, and the implicit times are prolonged [15]. Thus, the absence of the pikachurin protein disturbs the signal transmission from the photoreceptors to the post-receptoral components.

The purpose of this study was to determine whether post-receptoral interactions or the photoreceptor potentials were associated with the increase of the ERG during light adaptation. To answer this question, we recorded ERGs from Pikachurin null-mutant (Pika-/-) mice as a model of an ocular system with disruptions of the post-receptoral components. We shall show that the photopic b-wave did not increase but decreased by about 20% in Pika-/- mice. Our results indicated that Pikachurin is essential for the increase of the photopic b-wave during light adaptation. Additional experiments using pharmacological agents indicated that the extracted post-receptoral component but not photoreceptoral component was associated with this phenomenon.

## Methods

### Animals

All experimental procedures adhered to the ARVO Statement for the Use of Animals in Ophthalmic and Vision Research and the guidelines for the Use of Animals of the Nagoya University School of Medicine. The Nagoya University Animal Experiment Committee approved this project (Approval Number, 24317).

Fifty wild type (WT) C57BL/6 mice and 50 Pikachurin null mice (Pika-/-) at 8 to 10 weeks-of-age were studied. The Pika-/- mice were kindly given to us by Dr. Takahisa Furukawa of the Institute for Protein Research, Osaka University. The mice were maintained in a 12-h light (<40 lux) and12-h dark environment.

### ERG recordings

The procedures used were similar to those described in detail [17]. Briefly,mice were anesthetized with an intramuscular injection of ketamine and xylazine after at least two hours of dark adaptation. The pupils were dilated with topical 0.5% tropicamide and 0.5% phenylephrine HCl. The pupil diameters were similar (around 2 mm) in WT and Pika-/- mice.The mice were placed on a heating pad for the duration of the ERG recordings. ERGs were recorded with a gold wire loop electrode on the cornea and a gold wire reference electrode placed on the sclera. Hydroxyethyl cellulose was used for hydration of the cornea and conjunctiva and to ensure good electrical conductivity of the electrodes. Signals were amplified and band pass filtered between 0.3 and 1000 Hz. The ERGs were averaged using a computer-assisted signal averaging system (Power Lab; AD Instruments, Castle Hill, Australia). A ganzfeld bowl (Model GS2000; LACE Electronica sel via Marmiccilo, Pisa, Italy) with a xenon source was used for stimulation.

### Intensity-response functions of scotopic and photopic ERGs

For the scotopic ERGs, 2 to 10 responses were averaged with an interstimulus interval of 3 to 60 sec, and for the photopic ERGs, 20 to 30 responses were averaged with a repetition rate of 1 sec. The maximum luminance was 1.0 log cd-s/m^2^, and neutral density filters were used to reduce the stimulus intensity. Eleven equal stimulus intensity steps, ranging from −5.0 to 1.0 log cd-s/m^2^, were used to elicit the scotopic ERGs, and 9 equal stimulus intensity steps, ranging from −1.4 to 1.0 log cd-s/m^2^, were used to elicit the photopic ERGs on a rod-desensitizing white adapting halogen background light of 40 cd/m^2^.

### Photopic ERGs during light-adaption

The mice were dark-adapted for at least 2 h before the ERG recordings. Following electrode placement in dim red light, the mice were dark-adapted for an additional 5 min before beginning the ERG recordings. The first ERG recordings were made immediately after turning on the light adapting background light of 40 cd/m^2^. ERGs were averaged with a repetition rate of 1 sec for one session, and it took 30 seconds to record the ERGs for one time period. ERGs were then recorded every minute thereafter for nine minutes. In the end, 10 ERGs were recorded beginning at 0 to 9 min after the light-adaptation background light was turned on. Two stimulus intensities, 0.4 and 1.0 log cd-s/m^2^, were used to elicit the ERGs. For each ERG recording during light adaptation, one of the stimulus intensity was used.

### Drug injections

To evaluate the function of the photoreceptors and the post-receptoral components, L-2-amino-4-phosphonobutyric acid (APB, Sigma-Aldrich, St. Louis, MO) and cis-2,3-piperidine-dicarboxylic acid (PDA, Sigma-Aldrich, St. Louis, MO) or γ-aminobutyric acid (GABA)-A receptor antagonist (bicuculline: Sigma-Aldrich, St. Louis, MO) were injected intravitreally in the WT and Pika-/- mice. Mice were also injected with a mixture of APB and PDA solution (APB/PDA) or bicuculline in one eye and PBS in the fellow eye. The APB, PDA and bicuculline were dissolved in sterile saline, and the intravitreal concentration was estimated to be 1 mM, 5 mM and 100 μM respectively. [18,19] Drug concentration at the retina was calculated using an estimated vitreal volume of 5 μl in mice [20]. Mice were anesthetized with ether, and 1 μL of solution were injected intravitreally with a glass micropipette with a microinjection system (IM 300 microinjection; Narishige, Tokyo, Japan) [21]. In more detail, a sharpened tip of a micropipette was passed through the sclera, just posterior to the limbus into the vitreous cavity, and a foot switch was depressed. Injections were performed with a condensing lens system on the dissecting microscope and a plastic ring filled with gonioscopic solution, which allowed a view of the retina and avoiding hitting the lens. ERG recordings were begun approximately 2 hours after the drug injections.

### Extracted post-receptoral component

We determined the post-receptoral component based on the ERGs from the four groups of mice; Pika-/- mice injected with APB/PDA, Pika-/- mice injected with PBS, WT mice injected with APB/PDA, and WT mice injected with PBS. We averaged the ERGs recorded from each group of mice at each time point. Then, we isolated the post-receptoral component by subtracting averaged ERGs recorded from the APB/PDA injected eyes from the PBS injected eyes at each time point. The extracted components were taken to be the post-receptoral component.

### Statistics analyses

Repeated measures ANOVA was used to determine the significance of any differences in the thickness of the retinal layers and evaluation of the ERG amplitudes. A *P* <0.05 was considered significant.

## Results

### Intensity-response series of scotopic and photopic ERGs

**Fig 1:**
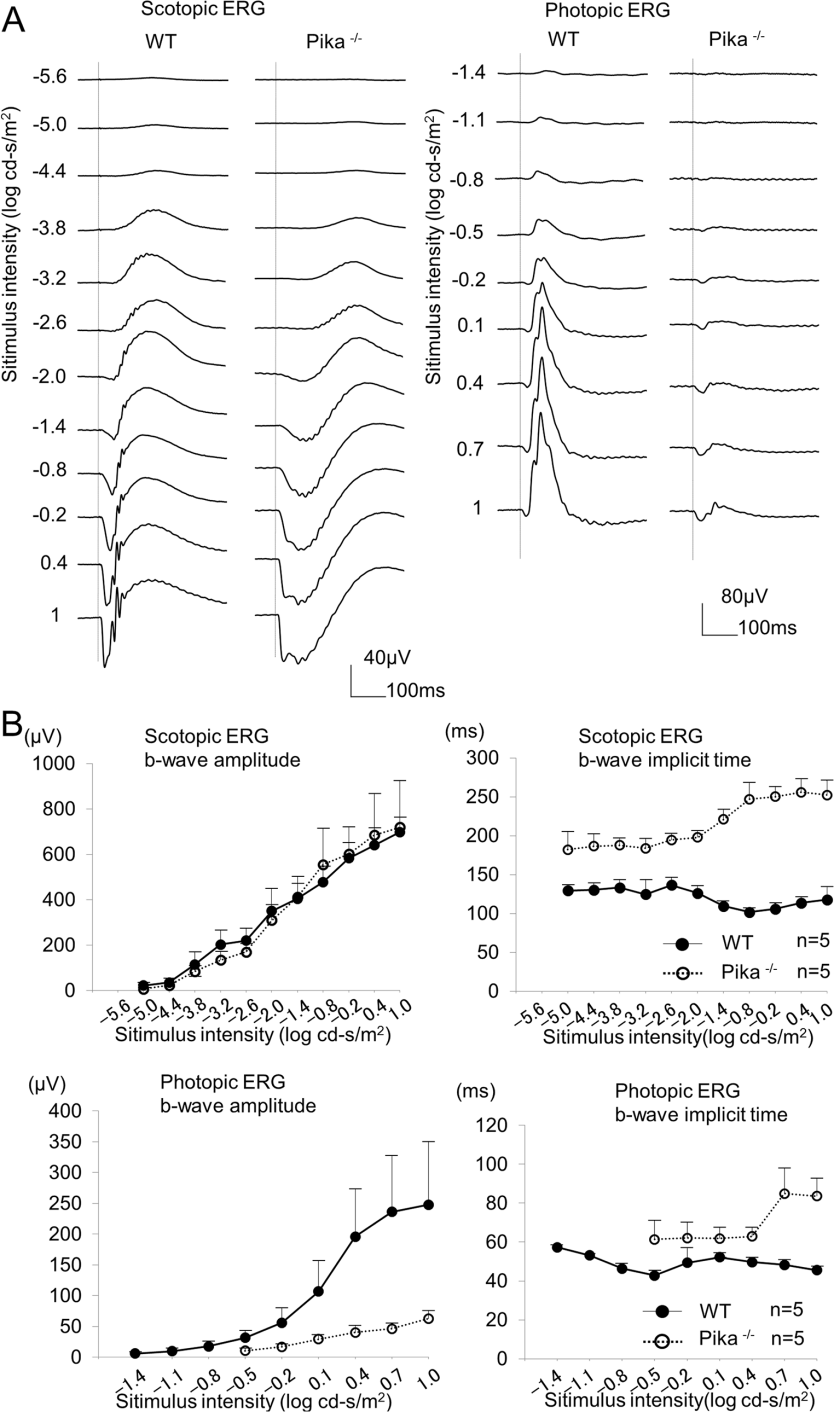
Effect of different stimulus intensities on the amplitudes of the a- and b-waves of the scotopic (A) and photopic (B) ERGs of wild type (WT) and Pika-/- mice. (A) Scotopic flash ERGs elicited by 11 different stimulus intensities (from -5.0 to 1.0 log cd-s/m^2^), and photopic flash ERGS elicited by 9 different stimulus intensities (from -1.4 to 1.0 log cd-s/m^2^) recorded in a representative WT mouse and a representative Pika-/- mouse. (B) The intensity–response curves of the mean scotopic and photopic b-wave amplitudes of five WT mice and five Pika-/- mice are shown in the upper left and lower left panels respectively. The amplitudes of the scotopic b-wave of WT mice were not significantly different from that of Pika-/- mice (P >0.05: repeated measures ANOVA). However, the amplitudes of the photopic b-wave of Wt mice were significantly different from Pika-/- mice (P <0.05: repeated measures ANOVA). In addition, the intensity–response curves of the mean scotopic and photopic b-wave implicit times are shown in the upper right and lower right panel respectively. The implicit times of the scotopic and photopic b-waves of WT mice were significantly different from Pika-/- mice (P <0.05: repeated measures ANOVA).The means ± SDs are plotted. ● = WT mice and ○ = Pika-/- mice.

To confirm the functional abnormality of signal transmission from the photoreceptors to the bipolar cells due to the absence of pikachurin, we recorded scotopic and photopic ERGs from WT mice and Pika-/- mice. The scotopic ERGs elicited by 13 different stimulus intensities from a WT mouse and a Pika-/- mouse are shown in Fig 1A. In both types of mice, only a positive b-wave, which originates mainly from the activity of the rod bipolar cells [22,23], was elicited at lower stimulus intensities (−5.6 to −2.6 log cd-s/m^2^). At higher stimulus intensities (−1.4 to 1.0 log cd-s/m^2^), a negative-going a-wave appeared, which originates mainly from the activity of the rod photoreceptors [24,25]. The amplitudes of both the a-wave (data not shown) and b-wave were not significantly different in the two types of mice. (Fig 1B) However, the implicit times of the b-wave were significantly delayed at all stimulus intensities in the Pika-/- mice (Fig 1B).

The photopic ERGs elicited by 9 different stimulus intensities from a WT mouse and a Pika-/- mouse are shown in Fig 1A. These ERGs were recorded 10 minutes after light-adaptation with a background light of 40 cd/m^2^. In contrast to the scotopic ERGs, the amplitudes of the photopic b-wave in the Pika-/- mice were significantly smaller than that of the WT mice. At the maximum stimulus intensity (1.0 log cd-s/m^2^), the b-wave amplitude of the Pika-/- mice was only 25% of that of the WT mice. The implicit times of the photopic b-waves were also significantly delayed in Pika-/- mice at all intensities (Fig 1B). Although the amplitudes of the photopic b-waves were severely reduced in Pika-/- mice, the oscillatory potentials still remained (Fig 1A). These results are consistent with the past ERG recordings of Pika-/- mice [15].

### Photopic ERG alterations during light-adaptation

**Fig 2:**
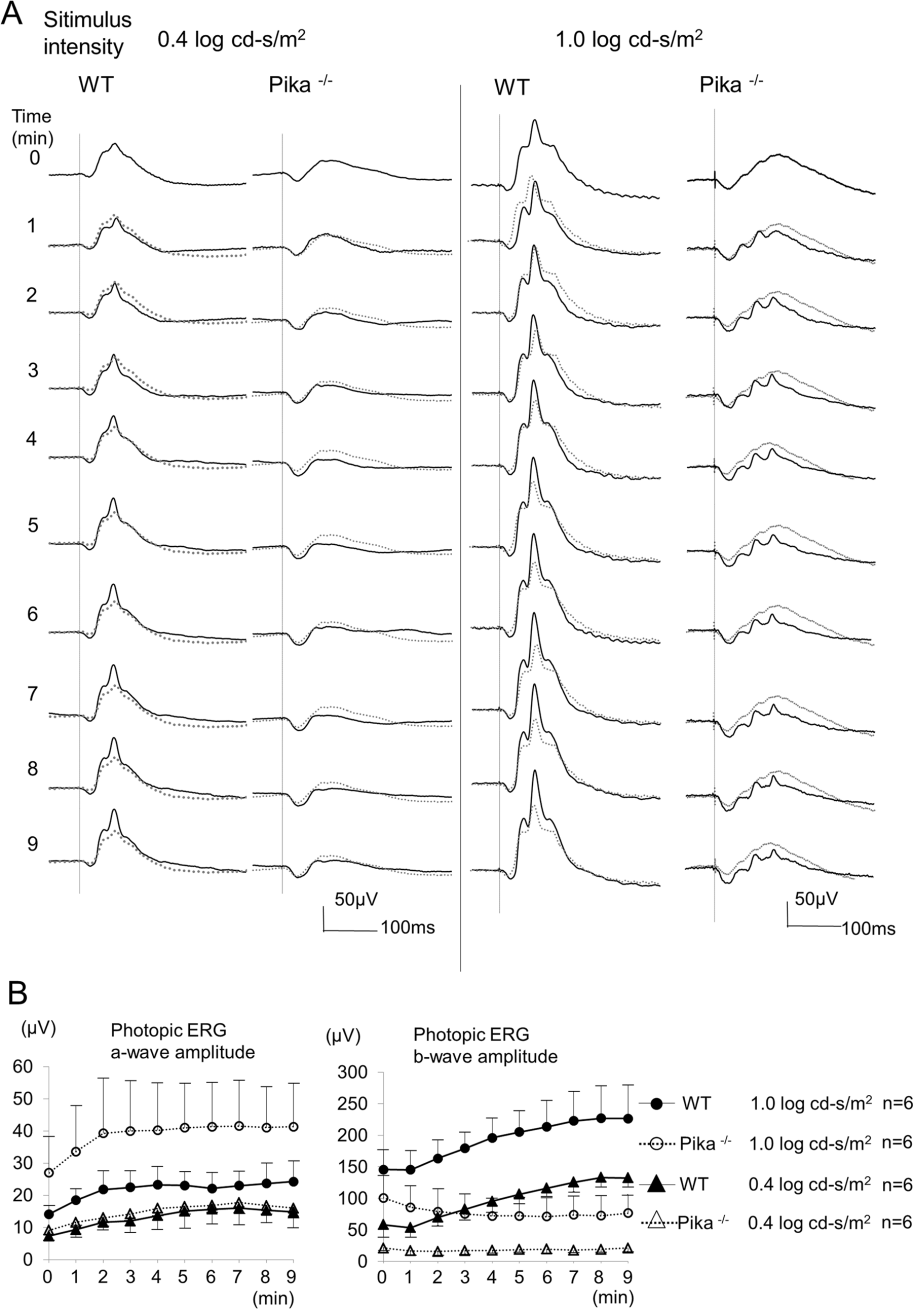
ERGs of wild type (WT) and Pika-/- mice during the light adaptation. (A) Photopic ERGs recorded at each minute for 9 minutes of light adaption (40 cd/m^2^) from a representative WT mouse and a Pika-/- mouse. ERGs were elicited by two different stimulus intensities, 0.4 and 1.0 log cd-s/m^2^. ERGs recorded at each minute for 9 minutes of light adaptation (black trace) are superimposed on the ERG elicited at the start of light adaptation (0 min). (B) Time-response curves of the mean a- and b-wave amplitudes of 5 WT mice and 5 Pika-/- mice are shown in the right and left panel respectively. The amplitudes of the photopic a- and b-waves during light-adaptation of WT mice were significantly different from that of Pika-/- mice (P <0.05: repeated measures ANOVA). ● = ERGs of WT mice recorded with 1.0 log cd-s/m^2^. ○ = ERGs of Pika-/- mice recorded with 1.0 log cd-s/m^2^. ▲ = ERGs of WT mice recorded with 0.4 log cd-s/m^2^. △ = ERGs of Pika-/- mice recorded with 0.4 log cd-s/m^2^. The means ± SDs are plotted.

Representative photopic ERGs recorded at each minute for 9 minutes of light-adaptation from a WT mouse and a Pika-/- mouse are shown in Fig 2A. The ERGs were elicited by stimulus intensities of 0.4 and 1.0 log cd-s/m^2^. For both stimulus intensities, the amplitudes of the photopic b-waves of the WT mouse gradually increased during light adaptation. On the other hand, the b-waves of the Pika-/- mice decreased during light-adaptation for both stimulus intensities. We recorded ERGs with a repetition rate of 1 and 5 sec, but the results were same. We chose to record photopic ERGs during the light adaptation with a repetition rate of 1 second.

The amplitude of the a- and b-waves of the photopic ERG elicited by two different intensities during light-adaptation in both type of mice are shown in Fig 2B. The amplitude of a-waves increased and reached plateau after 3–5 minutes of light adaptation in both type of mice with the two stimuli. The amplitude of the a-waves increased by 1.7 times for WT mice and 1.5 times for Pika-/- mice for stimulus of 1.0 log cd-s/m^2^, and by 2.0 times for WT mice and 1.8 times for Pika-/- mice for stimulus of 0.4 log cd-s/m^2^. These results indicated that the amplitudes of the a-waves increased during the course of light adaptation in both types of mice. The amplitudes of a-wave of Pika-/- mice were significantly larger than those of WT mice at all times during the light adaptation with stimulus of 1.0 log cd-s/m^2^. This larger a-wave in Pika-/- mice appeared to be due to an unmasking of the a-wave by the delay of the b-wave. But the amplitude of a-wave elicited by 0.4 log cd-s/m^2^ was not significantly different between the two types of mice probably because of its smaller amplitude.

The changes of the b-wave amplitudes during light-adaptation were different for the two types of mice. In the WT mice, the amplitudes decreased over the first minute and then began increasing and reached plateau after 7–8 minutes of light-adaptation for both stimulus intensities. The amplitude of the b-wave of the WT mice increased 1.5 times with stimulus of 1.0 log cd-s/m^2^ and 2.2 times with stimulus of 0.4 log cd-s/m^2^ relative to the initial amplitude. The slight reduction of the b-wave of WT mice in the first minute of light adaptation was similar to the reduction reported in human photopic ERGs [15].

In Pika-/- mice, the amplitude of the b-waves decreased for 3–4 minutes after the beginning of the light-adaptation and reached a plateau. The amplitudes of the photopic ERGs of Pika-/- mice, which were recorded nine minutes after beginning the light-adaptation, decreased by 0.8 times compare to that of initial ERG for both stimulus intensities.

### After injection of APB and PDA

To confirm that the amplitude reduction of the photopic ERG during light-adaptation in Pika-/- mice was caused by abnormalities of the post-receptoral components, we injected PBS or APB/PDA into the vitreous of WT mice and Pika-/- mice. APB is an inhibitor of metabotropic glutamate receptor, and this inhibition blocks transmission from the cones to the ON-bipolar cells [26]. PDA is a glutamate analog that blocks transmission from the cones to the OFF-bipolar cells and horizontal cells [27]. We aimed to determine the photoreceptor potential by inhibiting all of the post-receptoral components with the injection of APB/PDA. Then, we subtracted these waves from the ERGs recorded from eyes with PBS injection to extract the post-receptoral component. We determined which component, the photoreceptor component or the post-receptoral component, contributed to the ERG reduction in Pika-/- mice during light-adaptation. In these experiments, the ERGs were elicited by a stimulus intensity of 1.0 log cd-s/m^2^ because the former experiments indicated that the changes of the ERGs during light-adaptation elicited with 1.0 and 0.4 log cd-s/m^2^ stimuli had the same pattern.

**Fig 3:**
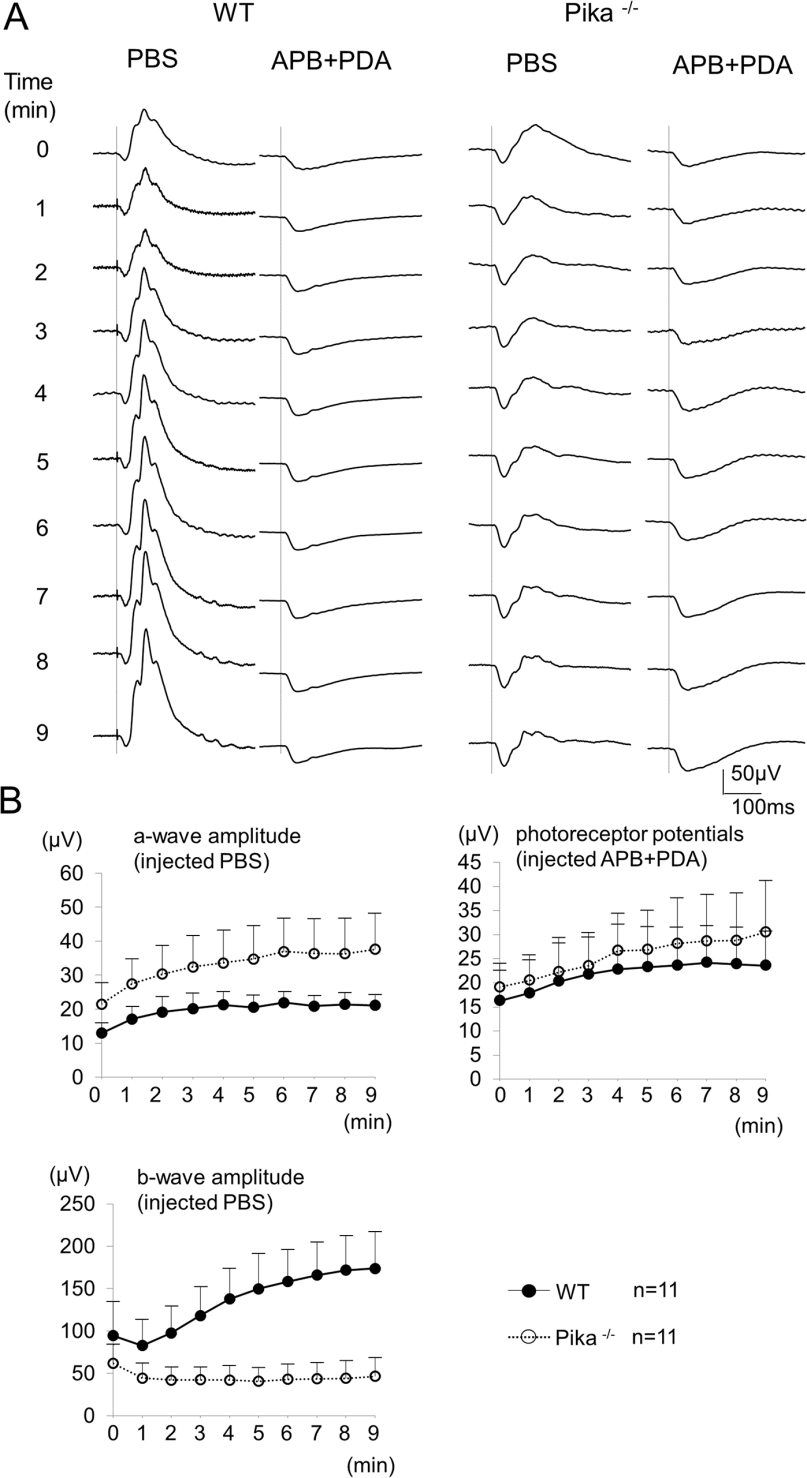
ERGs of wild type (WT) and Pika-/- mice after injection of PBS or APB/PDA. (A) Photopic ERGs series recorded at each minute for 9 minutes of light adaptation. The ERGs were elicited by a stimulus intensity of 1.0 log cd-s/m^2^ after injection of PBS or APB/PDA. The ERGs recorded from WT are shown on the left and those of Pika-/- mice are shown on the right. (B) Time-response curves of the mean amplitudes of the a- and b-waves of 11 WT mice and 11 Pika-/- mice after intravitreal injection of PBS. The curve for the WT mice is shown on the upper left and that for Pika-/- mice is shown on lower left respectively. Time-response curves of the mean amplitudes of a-wave of 11 WT mice and 11 Pika-/- mice after intravitreous injection of APB + PDA are shown in the upper right. Photopic photoreceptor potentials after the injection of APB+PDA during light adaptation were not significantly different between WT and PIKA-/- mice (P >0.05: repeated measures ANOVA). ● = ERGs of WT mice and ○ = ERGs of Pika-/- mice. The means ± SDs are plotted.

The photopic ERGs elicited at each minute for 9 minutes after an intravitreous injection of PBS into one eye and APB/PDA into the other eye of a wild-type mouse and those of Pika-/- mouse are shown in Fig 3A. The ERGs after PBS injection were slightly smaller than those of the uninjected eyes (Fig 2) for both types of mice. However, these ERGs had similar characteristics with those without injection (Fig 3A). The amplitude of a-waves increased during the light-adaptation by 1.5 times in the WT mice and 1.8 times in the Pika-/- mice relative to the initial wave. On the other hand, the amplitude of b-waves of Pika-/- mice after PBS injection decreased over the first 3–4 minutes after light adaptation, and the amplitudes decreased by 0.8 times than that of the initial b-wave. After the injection of APB/PDA, the positive wave completely disappeared, and a slow negative component remained in both types of mice. The waveforms of these two types of mice were comparable. The negative wave represented the contribution from the photoreceptors. The amplitude of the negative a-wave of both wild-type and Pika-/- mice increased during light adaptation. The a-wave increased 1.4 times in WT mice and 1.6 times in the Pika-/- mice relative to the initial wave. The amplitude of a-waves was not significantly different between the two types of mice during the course of light adaptation (Fig 3B). These results indicated the same photoreceptor components were included in ERG of the both types of mice.

### Post-receptoral component

**Fig 4:**
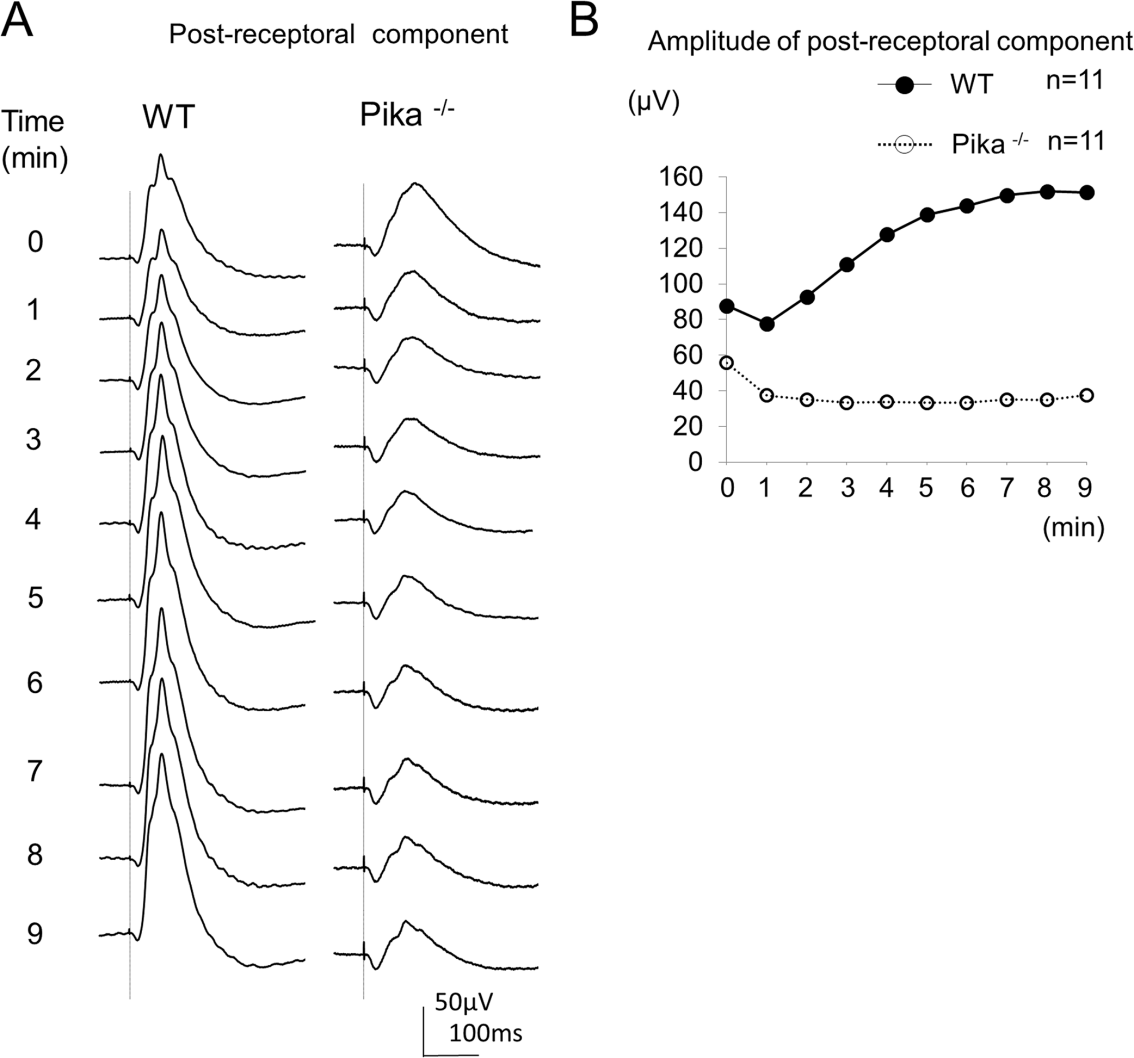
Extracted post-receptoral components of WT and Pika-/- mice. (A) Waves were obtained by subtracting the averaged ERGs of the 11 eyes injected with APB + PDA from those of 11 eyes injected with PBS. The subtracted waveforms at each minute for 9 minutes of light adaptation of WT mice and Pika-/- mice, which indicated post-receptoral component, are shown. (B) Amplitudes of maximum positive wave of the subtracted waveforms are plotted during the course of light adaptation of WT mice and Pika-/- mice.

Next, we extracted the post-receptoral component from the ERGs obtained after the injection of PBS or APB/PDA. The results of the ERGs recorded from 11 WT mice and 11 Pika-/- mice injected PBS or APB/PDA were averaged. The waveforms obtained by subtracting the averaged ERGs of the eye injected with APB/PDA from those injected with PBS are shown in Fig 4A.These waveforms represent the contribution of the post-receptoral component to the photopic ERGs during light-adaptation. We measured the amplitude of the maximum positive waves of the subtracted waveforms that resembled the photopic b-wave of the two types of mice. (Fig 4B)

The amplitude of the positive wave of the WT mice decreased over the first minute, then increased and reached plateau after 7–8 minutes of light-adaptation. The amplitude of the WT mice increased by1.7 times relative to the initial amplitude. The amplitude of the maximum positive waves in Pika-/- mice kept decreasing and reached a plateau after 3–4 minutes of light-adaptation. The amplitude of the Pika-/- mice decreased by 0.7 times relative to the initial amplitude.

### Effect of GABA-A receptor antagonist

**Fig 5:**
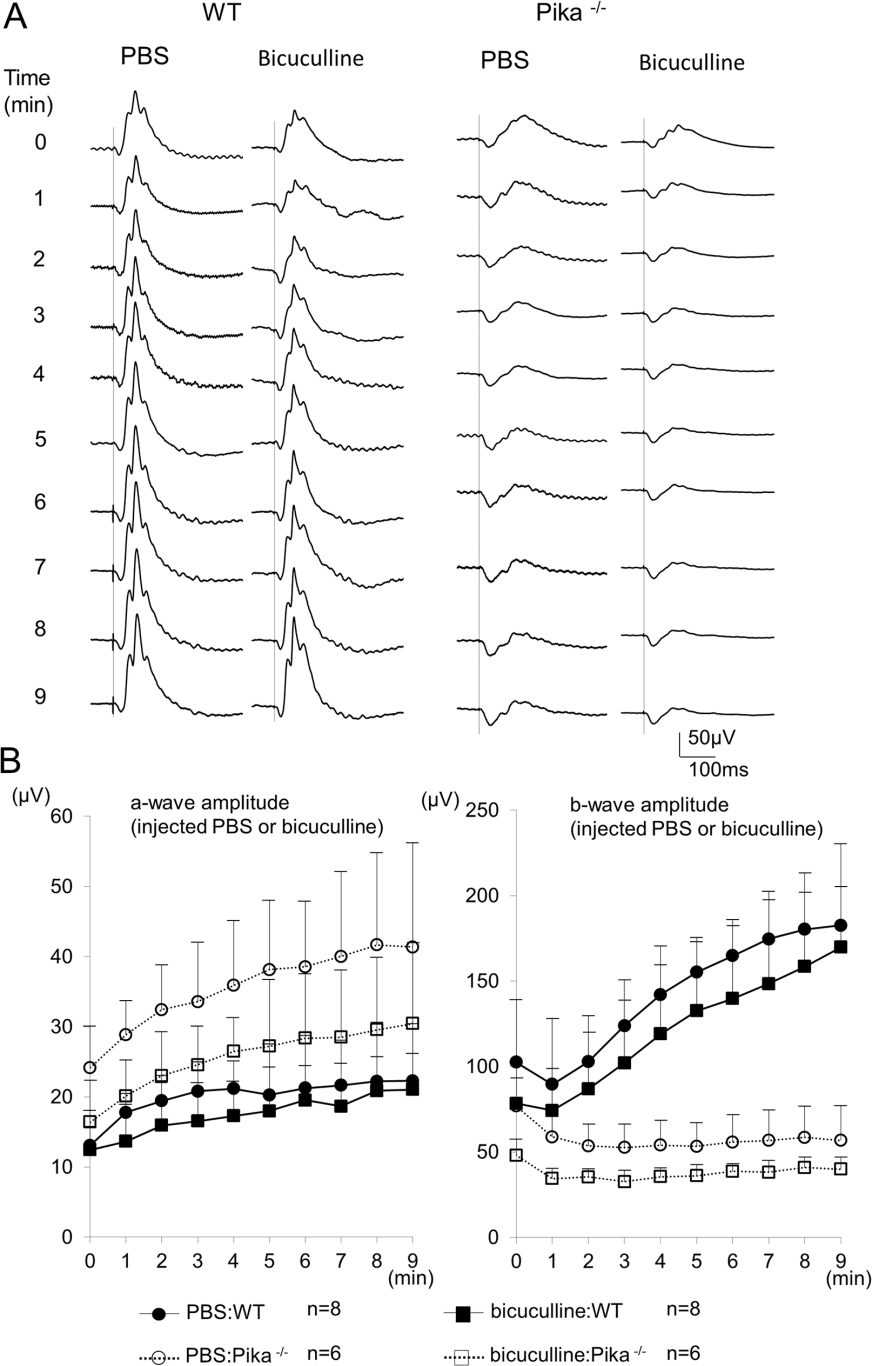
ERGs of WT and Pika-/- mice after injection of PBS or bicuculline. (A) Photopic ERGs series recorded at each minute for 9 minutes of light adaptation. ERGs elicited by stimulus intensity of 1.0 log cd-s/m^2^ after injection of PBS or bicuculline. The ERGs recorded from WT are shown on the left and those of Pika-/- mice are shown on the right. (B) Time-response curves of the mean amplitudes of the a- and b-waves of 8 WT mice and 6 Pika-/- mice after intravitreal injection of PBS or bicuculline are shown in the right and left panel respectively. In Pika-/- mice, the amplitudes of the photopic b-wave were significant different during light adaptation during light adaptation between PBS and bicuculline injection groups (P <0.05: repeated measures ANOVA). ● = ERGs of WT mice injected with PBS. ○ = ERGs of Pika-/- mice injected with PBS. ■ = ERGs of WT mice injected with bicuculline. □ = ERGs of Pika-/- mice injected with bicuculline. The means ± SDs are plotted.

To determine the mechanisms of the ERG reduction during light adaptation in Pika-/- mice, we injected a GABA-A receptor antagonist, bicuculline, into the vitreous of WT mice and Pika-/- mice. Horizontal cells are known to release GABA when they are depolarized [28], and the released GABA affects cone photoreceptors and cone ON bipolar cells through GABA-A receptors [13]. The photopic ERGs elicited at each minute for 9 minutes after an intravitreal injection of bicuculline into one eye and PBS into the other eye of a WT mouse and a Pika-/- mouse are shown in Fig 5A. The amplitude of the a- and b-waves after bicuculline injection were smaller than those of PBS injection during the course of light adaptation in both types of mice (Fig 5B). However, there were no significant differences in the amplitudes of the a-waves in both types of mice and in the b-waves in the WT mice (P <0.05: repeated measures ANOVA). The amplitude of b-waves of the Pika-/- mice during light adaptation were significantly smaller from those of the PBS-injected mice (P <0.05: repeated measures ANOVA). However, the amplitude decreased by 0.8 times more than that of the initial b-wave, which is almost same as that of PBS, and bicuculine did not seem to be related to the increase in the photopic b-wave of the WT and the reduction in the Pika-/- mice during light adaptation.

## Discussion

We used Pika-/- mice because they have reduced signal transmission between the photoreceptors and ON bipolar cells. This abnormality allowed us to examine the contribution of post-photoreceptor components to the increase of the photopic ERGs during light adaptation. The findings in the Pika-/- mice demonstrated that the photopic b-wave did not increase but decreased by about 20% in the first 3 to 4 minutes of light-adaptation and then plateaued. Additional experiments of the post-receptoral responses isolated by pharmacological agents confirmed that the extracted post-receptoral component decreased during the light-adaptation in the Pika-/- mice. However, the photoreceptoral component increased as they did in WT mice.

We draw two conclusions. The normal apposition of the photoreceptor synaptic zone and the retinal ON bipolar cell dendrites which is controlled by pikachurin is essential for the increase of the ERGs during light adaptation. And second, although several studies suggested that this ERG increase was caused by an increase of the cone photoreceptor potentials, the ERG increase cannot be explained by an increase of only the photoreceptoral potential because WT and Pika-/- mice showed similar photoreceptor potentials increase during light adaptation.

When we evaluated the changes of the amplitudes of the ERGs during the course of light adaptation, we found that the amplitude of the b-wave in WT mice decreased slightly over the first minute of light-adaptation as reported in humans [14]. Other data using flicker ERGs in frogs or cone ERGs in rats showed that the amplitude increase did not begin until after the first minute of the light-adaptation [10,12,29]. This slight reduction in the first minute seems to be common in different species. From our results using pharmacological agents, this reduction in the first minute was probably caused by a post-receptoral component. In the WT mice, the post-receptoral component decreased in the first minute and then increased, but in the Pika-/- mice the post-receptoral component gradual decreased for 3–4 minutes after the beginning of light-adaptation and did not increase. We suggest that the Pika-/- mice lack the post-receptoral component which increases after the first minutes of light-adaptation in WT mouse.

The results of earlier studies suggested that under dark-adapted conditions, rod activities inhibit cone responses, and this inhibition may be released under light-adapted conditions. This would then result in an increase in the cone ERGs [4,8,11]. If this is correct, our results support the hypothesis that the rod inhibition of cones through post-receptoral interaction causes this phenomenon [4,8,13].

Rod-cone interactions are essential for the adaptation of photoreceptor sensitivity to different levels of background light [30–32]. However, the synaptic pathways in the retina are complex, and only part of the mechanism of rod-cone interaction has been determined. For example, some studies showed that rods make chemical synapses with OFF cone bipolar cells [32,33], while another study showed that a certain type of ON cone bipolar cell formed ribbon-associated synapses not only with cones but also with rods [34]. In addition, horizontal cells play important roles in rod-cone interactions, and they receive a mixture of rod and cone inputs and integrate the information of both pathways [32]. Jackson et al. showed that in dopamine D1 receptors knock out mice, the increase of the b wave amplitude during light adaptation is less [35]. Dopamine D1 receptors modulate horizontal cells [36,37], and the dopamine induces GABA release via D1 receptors [38]. Because cone photoreceptors and cone ON bipolar cells possess GABA–A receptors, GABA is supposed to be related with feedback and feed-forward pathways in horizontal cells. Our results showed that bicuculline did not affect Pika-/- mice or WT mice which would indicate that the change of the ERGs during light adaptation was not related with GABA-A receptor activation. This suggested the possibility that a horizontal cells through GABA release do not play significant roles in the increase in the b-waves of the cone ERGs during light adaptation. From our results, it is difficult to determine which rod-cone interaction in the post-receptoral component was eliminated by the knock-out of pikachurin. To determine this more accurately, we need to determine the alterations of the post-receptoral interactions between rods and cones caused by the absence of pikachurin in more detail.

As best we know, there have been no studies that reported a reduction of the ERGs during light adaptation in human diseases or animal models. To analyze the ERG of Pika-/- mouse during light adaptation revealed a part of underlying mechanisms for the increase of ERGs during light-adaptation.
